# Stage-specific immune responses in human *Necator americanus* infection

**DOI:** 10.1111/j.1365-3024.2007.00950.x

**Published:** 2007-07

**Authors:** S M GEIGER, I R CALDAS, B E MC GLONE, A C CAMPI-AZEVEDO, L M DE OLIVEIRA, S BROOKER, D DIEMERT, R CORRÊA-OLIVEIRA, J M BETHONY

**Affiliations:** 1 Fundação Oswaldo Cruz, Centro de Pesquisas René Rachou Belo Horizonte-MG, Brazil; 2 The George Washington University Medical Center, Department of Microbiology, Immunology, and Tropical Medicine Washington, DC, USA; 3 London School of Hygiene and Tropical Medicine, Departments of Infectious and Tropical Diseases, and Epidemiology and Population Health London, WC1E 7HT, UK; 4 Albert B. Sabin Vaccine Institute Washington, DC, USA

**Keywords:** *cytokines*, *hookworm*, *immune response*, Necator americanus, *principal components analysis*, *T cells*

## Abstract

*We describe how hookworms interact with their human hosts by comparing lymphocyte phenotyping, proliferative responses, and cytokine and chemokine secretion patterns in adults who are either mono-infected with* Necator americanus *or egg-negative controls resident in an area of high transmission in Brazil. Cellular immune responses against crude hookworm antigen extracts from different developmental stages were evaluated simultaneously. Principal component analysis (PCA) was used to reduce the standardized immune responses. Random effects multivariate regression was then used to investigate whether principal components (PC) differ between the two groups once potential confounders and effect modifiers have been accounted for. Although hookworm patients had reduced percentages of T and B cells, they had higher levels of activated CD4^+^ T and CD19^+^ B cells. This state of ‘immune activation’ coincided with lower proliferative responses, especially to third-stage larval antigen. Cytokine levels in mono-infected adults were also lower and characterized by a mixed Th1/Th2-type profile. Excretory/secretory antigen from adult worms was a potent modulator of the immune response, resulting in diminished TNF-α and IL-10 secretion in peripheral blood mononuclear cells (PBMC) from hookworm infected patients. We propose that the longevity of hookworms in their human hosts results from a stage-specific, down-modulation of the immune response*.

## INTRODUCTION

In contrast to the other major soil-transmitted helminths *Ascaris lumbricoides* and *Trichuris trichiura*, where peak intensity of infection occurs in young children, the intensity of hookworm infection is highest among older children and adults (1–3), with the elderly often harbouring the heaviest worm burdens (4,5). This age-intensity profile, coupled with several years of longevity of adult hookworms in the host (6), suggests that this parasite is refractory to host immune responses. Moreover, not only do hookworms ward off immune attack, they may also modulate the immune response from the most damaging modes to ones that are more beneficial for successful residence, feeding and reproduction (7).

Recent immunoepidemiological studies of hookworm suggest that, in contrast to the skewed T-helper 2 (Th2) response characteristic of most helminth infections (7), hookworms elicit a ‘mixed’ Th1/Th2 cell response in which both Th1 (interferon (IFN)-γ and IL-12) and Th2 (IL-4, IL-5 and IL-13) cytokines are produced (8–10). There is evidence for the importance of a Th2 response during human hookworm infection, with a significant association reported between IL-5 secretion and reduced hookworm burden (10). Combined with studies from laboratory animals (11,12), these findings add to the mounting evidence that a Th2-immune response is protective against hookworm.

Hookworm infection is also accompanied by elevated levels of background and hookworm antigen-specific IL-10, a regulatory cytokine, and down-regulation of antigen-specific and nonspecific cellular reactivity (3,9). The precise mechanisms involved in the down-regulation of T-cell reactivity during hookworm infection are poorly understood. A recent study indicated that infected individuals have markedly diminished percentages of CD3^+^ and CD4^+^ T-cells (13). In contrast to lower T-cell counts, the immune response in individuals infected with multiple different helminths has also been characterized as a ‘highly activated’ phenotype, with marked increases in CD3^+^/HLA-DR^+^, CD4^+^/HLA-DR^+^ and CD8^+^/HLA-DR^+^ T-cells, together with a diminished number of CD4^+^ naïve and increased number of CD4^+^ memory T-cells (14,15). These changes in lymphocyte activation are accompanied by increased levels of total and antigen-specific immunoglobulins, particularly IgG4 and IgE, and by eosinophilia (16,17).

In this study we investigated differences in the lymphocyte subpopulations between egg-negative and hookworm mono-infected individuals living in an area of Brazil endemic for *Necator americanus*, as well as their proliferative and cytokine responses to antigens from different developmental stages of hookworm. Stage-specific immune responses were examined in an attempt to further elucidate the mechanisms by which the hookworm parasite interacts with its human host.

## MATERIALS AND METHODS

### Field surveys

The study was conducted in 2004 in Americaninhas, Municipality of Novo Oriente, in the state of Minas Gerais, Brazil. Details of the study area and population are reported elsewhere (18). Presence of infection was determined by formalin–ether sedimentation and, if positive, two more stool samples were analysed by the Kato–Katz faecal thick-smear technique (19) and parasite load was expressed as eggs per gram of faeces (epg). Individuals found to be infected with hookworm were treated with albendazole (400 mg). Faecal samples from a subset of infected individuals were obtained following treatment and examined for expelled worms. Worms were clarified in a 50% phenol solution and their buccal capsules analysed under a light microscope (100–400× magnification); of 150 male and female adult worms examined, all were identified as *N. americanus*.

Two-hundred and fifty adults were selected by simple random sampling for immunological assays. From these, data from 27 egg-negative and 28 hookworm mono-infected adults were included in the present study. All other patients were infected with other helminths or coinfected with two or more helminth species. Approximately 20 mL of blood was collected in heparinized tubes for separation of peripheral blood mononuclear cells (PBMC) and 4 mL of blood in EDTA tubes for the immunological assays described below. The study was approved by the ethical review committees of The George Washington University (GWU, USA), the London School of Hygiene and Tropical Medicine (UK), the Centro de Pesquisas René Rachou FIOCRUZ and the Brazilian National Committee for Ethics in Research (CONEP).

### Parasite antigen preparation

The experiments were conducted according to a protocol approved by the GWU Animal Care and Use Committee. *Ancylostoma caninum*L3 and adult worms were obtained from purpose-bred, male beagles (Marshall Farms) maintained at the GWU Animal Research Facility as previously described (20). Adult worm ES products were prepared according to Carr and Pritchard (21) and stored in aliquots at –80°C. All antigen preparations used in cell cultures were passed through a 0·22 µm low-protein binding syringe filter (Millipore) and the resulting protein concentration was determined by using the BCA protein assay kit (Pierce).

In order to eliminate any possible stimulatory effects in tissue culture from LPS in crude *A. caninum* antigen preparations (L3, AE and adult ES), crude antigen preparations were assessed for LPS content using the Sigma E-TOXATE assay kit in accordance with the manufacturer's instructions. No LPS was detected in any of the *A. caninum*crude antigen extracts used in this study.

### Lymphocyte phenotyping

Two-colour phenotyping of lymphocytes was done for the evaluation of membrane expression of activation markers, markers of memory and co-stimulatory molecules. Monoclonal antibodies (mAb) conjugated with either phycoerythrin (PE) or fluorescein isothiocyanate (FITC) were used. Briefly, 100 µL of EDTA blood samples were incubated with 10 µL of mAb in the dark at room temperature (RT) for 30 min, lysed, washed twice in PBS (pH 7·2), and re-suspended in 500 µL 1% formaldehyde/PBS. The following pairs of mAb were used: CD4 (FITC)/CD25 (PE), CD4 (FITC)/HLA-DR (PE), CD4 (FITC)/CD45RO (PE), CD4 (FITC)/CD45RA (PE), CD8 (FITC)/CD28 (PE), CD8 (FITC)/HLA-DR (PE), CD8 (FITC)/CD45RO (PE), CD8 (FITC)/CD45RA (PE), CD3 (FITC)/CD69 (PE) and CD19 (FITC)/CD27 (PE). Cells incubated with FITC- or PE-conjugated mouse IgG1 served as isotype controls.

### *In vitro* proliferation of lymphocytes

Separation of PBMC was performed as described elsewhere (22). In 96-well cell culture plates, triplicates of 250 000 or 150 000 cells/well were added for antigen and mitogen stimulations, respectively, in a final volume of 200 µL complete RPMI-1640 (22). Final concentrations of stimulants determined to be optimal in cell culture were: 35 µg/mL for L3, AE and ES antigens, and 2·5 µg/mL for phytohaemagglutinin (PHA)-P (Difco Laboratories, Detroit, MI, USA). Cells were cultured at 37°C in a humidified 5% CO_2_incubator. Tritiated thymidine (Amersham Pharmacia, São Paulo, Brazil; 0·5 µCi/culture; specific activity 6·7 Ci/mm) was added to the cultures at 48 h (mitogen stimulation) or 120 h (antigen stimulation) and cells were harvested 18 h later. Incorporated tritiated thymidine was determined in a liquid scintillation counter and the data expressed as stimulation indices (SI) (mean proliferation of stimulated culture divided by mean proliferation of unstimulated culture).

### Cytokine and chemokine detection in cell culture supernatants

For production of cytokines and chemokines, 5 × 10^5^ PBMCs were cultivated in 48-well tissue culture plates (Costar, Corning, NY, USA) at a total volume of 400 µL in complete RPMI-1640 for 2 (mitogen stimulation), 4 and 6 days (antigen stimulation), using the same final mitogen and antigen concentrations as described above. Cell-free supernatants were stored at –70°C until cytokine/chemokine quantification. A BD™ Cytometric Bead Array kit (CBA, BD Biosciences, San Diego, CA, USA) was used to detect concentrations of IFN-γ, TNF-α, IL-10, IL-5, IL-4 and IL-2 in 4-day cell culture supernatants stimulated with hookworm antigens and in unstimulated control cultures. With some modifications to the manufacturer's protocol, 25 µL of each sample were diluted 1 : 5 in assay diluent. In parallel, ninefold serial dilutions were performed with the provided standard in order to obtain a standard curve within the range between 20 and 5000 pg/mL. Assay diluent alone served as a negative control. A 15 µL of mixed cytokine capture beads were added and the samples incubated at RT in the dark for 90 min. Samples were washed with 500 µL of washing buffer and centrifuged for 7 min at 600 *g* and 18°C. After discarding the supernatants, beads were incubated with 18 µL of mixed PE-conjugated anti-human cytokine antibodies at RT for another 90 min in the dark. Beads were washed again (see above), re-suspended in 250 µL of washing buffer and immediately analysed using a facscan™ flow cytometer and the BD CBA Analysis Software (BD Biosciences). Results were expressed in pg/mL and the detection limits were as follows: 2·6 pg/mL for IL-2 and IL-4, 2·8 pg/mL for IL-10 and TNF-α, 2·4 pg/mL for IL-5 and 7·1 pg/mL for IFN-γ. Samples with cytokine concentrations greater than the standard were repeated at 1 : 20 or 1 : 50 dilutions by conventional sandwich ELISA (see below).

Antigen-induced secretion of IL-13 and the chemokine CXCL10 were detected in 4-day supernatants by ELISA. Additionally, because of differential cytokine kinetics, IFN-γ, IL-10 and IL-5 secretion were measured in 6-day supernatants from control and antigen-stimulated cultures by ELISA; mitogen-induced secretions of CXCL10, IFN-γ, TNF-α, IL-13, IL-10, IL-5, IL-4 and IL-2 were determined in supernatants from 2-day cultures also by ELISA (all ELISAs, R&D Systems, Minneapolis, MN, USA). When necessary, samples were diluted with PBS in order to obtain a value within the range of the standard curve. ELISAs were performed according to the manufacturer's protocols, using a total volume of 25 µL per well in high-binding half-area plates (Costar). On each plate, serial dilutions of standards were run to construct standard curves with the following ranges of concentration: IL-13 (23·4–3000 pg/mL); CXCL10 (15·6–2000 pg/mL); IFN-γ (7·8–1000 pg/mL); TNF-α (7·8–1000 pg/mL); IL-10 (23·4–3000 pg/mL); IL-5 (11·7–1500 pg/mL); IL-4 (15·6–2000 pg/mL) and IL-2 (7·8–1000 pg/mL). The sensitivity was 40 pg/mL for IL-13, whereas for all other ELISAs, the sensitivity was lower than the last standard dilution. The colourimetric reaction was read in an automated ELISA reader at 450 nm. Back calculations of cytokine concentrations from mean optical density values were extrapolated from the standard curves by using a 4-parameter curve fitting program (SOFTmax® pro 3.1.2).

### Statistical methods

All analyses were performed using Intercooled Stata 9.2 (Stata Corp, LP, USA). Examination of the data showed no impossible or implausible values, however, inspection of standardized residuals showed a number of immune responses that appeared to be outliers. These high values were not errors but a real reflection of the variation in immune responses and hence were included in the analysis. For missing immunological data, it was assumed that these intended measurements were missing completely at random (MCAR); data were missing from some time points for some individuals due to an insufficient number of PBMCs to perform all assays. Initial summary statistics were produced for demographic, parasitological and immunological data, with differences in demographic factors between groups assessed by chi-squared tests. Univariate and bivariate distributions of immunological responses to each antigen were also examined, as well as Spearman's rank pairwise correlation coefficients.

Due to the large number of immunological variables assayed and the correlation between some of the variables, principal component analysis (PCA) was used to reduce the standardized immune responses to just two or three principal components (PC); uncorrelated, linear combinations of the immunological variables. Since each PC can be thought of as a rotation of the original dimensional co-ordinate system, with the immunological variables as the co-ordinate axes, each PC measures different dimensions of the data with the maximum amount of variability. If the first few PC account for a substantial proportion of the variation in the data, they can be used to summarize the data with little loss of information. The number of PC retained was based on the proportion of the total variance explained by each PC as well as inspection of scree plots of eigenvalue size and number.

PC scores were derived by substituting an individual's immune response into each PC. The relative weight that each variable gave to the overall PC score (also known as loadings) and the correlations between immune response and component were examined to measure the contribution of each immunological variable to each PC (23,24) and hence used to determine variables that influence any differences in immune response between egg-negative and mono-infected individuals. If all weights were about equal then the PC was interpreted as an average of all the variables, whereas if some weights were small the corresponding variables were ignored and the PC interpreted in terms of a linear combination of a subset of the original variables.

PCA was applied separately to lymphocyte phenotyping as well as cytokine/chemokine responses after *in vitro* stimulation with each of the different antigens. PCA does not require a multivariate normal distribution but depends solely on the correlation matrix of the standardized immune responses (23); therefore, although immune responses, and in particular cytokine responses, are positively skewed, we did not transform the data to reduce skewness and improve normality. In addition, no single suitable transformation could be found which reduced the extent of skewness and improved normality.

PC scores and SI were then analysed using random effects regression (linear mixed models) to determine whether there were any significant differences in major peripheral blood lymphocyte populations, cytokine responses, and proliferation of lymphocytes between mono-infected and egg-negative individuals. Random intercept models were fitted using maximum likelihood to account for the clustering of individuals (level 1) within households (level 2), with random intercepts at the household level. Age and sex were fitted as fixed effects individually and then adjusting for each other, if found significant, to determine which factors may confound the effect of infection status on immune response. Interactions of the covariates with infection status were also fitted, if appropriate, to assess whether the covariates were modifying the effect of infection status on immune response. Due to the skewness in cytokine/chemokine and proliferative responses, corresponding 95% confidence intervals and significance levels for the mean differences were calculated by bootstrapping using accelerated bias correction (10 000 replicates) (25).

## RESULTS

### Demographic data and parasitological profile

Demographic characteristics of the 55 adults included in the study are detailed in Table 1. No differences were found between the egg-negative and mono-infected groups in the proportion of individuals in each age group, although there was a significantly higher proportion of males in the mono-infected group (*P* = 0·02). Accordingly, all subsequent analyses were adjusted for sex. In mono-infected individuals, the mean intensity of hookworm infection was 3641 epg of faeces (range 12–25 698).

**Table 1:** Demographic characteristics for adults with a *Necator americanus* mono-infection and egg-negative individuals living in Americaninhas, Minas Gerais State, Brazil

Characteristic		Mono-infected (*n* = 28)		Egg-negative (*n* = 27)	
			
Characteristic		Number	%^a^	Number	%^a^
Sex	Male	18	64	9	33
	Female	10	36	18	67
Age (years)	18–44	8	29	9	33
	45–64	12	43	9	33
	65+	8	29	9	33
Intensity of infection^b^ (epg)^c^	Light (< 2000)	20	71	NA	NA
	Moderate (2000–3999)	2	7	NA	NA
	Heavy (= 4000)	6	21	NA	NA

Notes: NA, not applicable

a Percentages may sum to more/less than 100 due to rounding

b Intensity of infection thresholds as proposed by WHO

c epg, eggs per gram of faeces.

### Lymphocyte cell subset characterization

**Figure 1:**
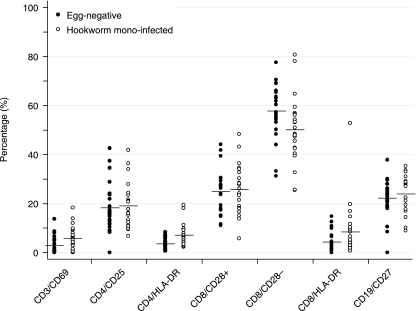
Percentages of lymphocyte populations expressing activation markers and co-stimulatory molecules on T-(CD3) and B-(CD19) cell populations and T-cell subpopulations (CD4 and CD8) among hookworm mono-infected and egg-negative individuals. Dots represent individual values and indicate the range and the bars indicate the mean percentages.

Hookworm-infected individuals had lower mean percentages of CD3^+^ (66%), CD4^+^ (35%) T-cell and CD19^+^ (9%) B-cell populations compared with egg-negative individuals (71%, 42% and 11%, respectively). However, expression of CD69 on CD3^+^ cells, HLA-DR on CD4^+^ and CD8^+^ cells, and CD27 on CD19^+^ cells were elevated in infected compared to egg-negative individuals (Figure 1). There was little difference in the percentage of CD8^+^ T-cells (33% infected; 32% egg-negative) between the two groups; however, the percentage of CD8^+^/CD28^−^ cells was lower among mono-infected individuals (50%) compared with egg-negative individuals (58%) (Figure 1). These observed differences are reflected in three PC which together explained 68% of the total variation in the data. Table 2 shows the relative weight that each of the major lymphocyte populations, their activation markers and co-stimulatory molecules give to each PC.

**Table 2:** Outcome of principal component analysis of the major peripheral blood lymphocyte populations and their expression of surface markers for activation, co-stimulation and regulation

	Principal component^a^^b^		
	
Lymphocyte population	1	2	3
CD3^+^	–0·142	**–0·460**	0·073
CD3^+^/CD69^+^	–0·019	**0·340**	0·266
CD4^+^	**0·371**	**–0·307**	0·027
CD4^+^/CD25^+^	0·167	**0·386**	0·035
CD4^+^/HLA-DR^+^	–0·287	**0·370**	–0·081
CD8^+^	**–0·495**	–0·151	0·052
CD8^+^/HLA-DR^+^	–0·086	**0·413**	0·209
CD8^+^/CD28^−^	**0·398**	0·247	0·143
CD8^+^/CD28^+^	**0·517**	–0·107	–0·002
CD19^+^	0·197	0·134	**–0·612**
CD19^+^/CD27^+^	0·123	–0·093	**0·688**

Notes:

a The figures represent the relative loading that each response contributes to each component. Strong loadings are indicated in bold

b Principal components 1 to 3 explained 68% of the total variation in major peripheral lymphocyte populations and the expression of cell surface markers.

After adjusting for sex, PC1 (Table 2) shows that mono-infected individuals had a significantly lower mean score (1·33; 95% CI 0·31–2·35; *P* = 0·01) than egg-negative individuals, that is, mono-infected individuals had a lower percentage of CD4^+^ T cells but elevated levels of HLA-DR on these CD4^+^ cells. Conversely, mono-infected individuals had a significantly higher percentage of CD8^+^ cells, with lower levels of CD28^+^ or CD28^−^ cells than egg-negative individuals.

PC2 (Table 2) reflects a contrast in the percentage of total CD3^+^ and CD4^+^ T-cells with the expression of early and late activation markers on these cells (CD3^+^CD69^+^; CD4^+^HLA-DR^+^) as well as the expression of HLA-DR on CD8^+^ T-cells. It is estimated that the mean score for PC2 among mono-infected individuals was 1·09 higher (95% CI 0·22–1·96) than that of egg-negative individuals. Regression analysis provides evidence that in mono-infected individuals the expression of early and late activation markers on CD3^+^ and CD4^+^ T-cells, as well as the expression of HLA-DR on CD8^+^ T-cells were significantly higher (*P =* 0·01), with lower percentages of total CD3^+^ and CD4^+^ T-cells when compared with egg-negative individuals.

PC3 contrasts CD19^+^ B cells with B cells expressing the activation marker CD27. The mean PC3 score was significantly higher (*P* = 0·02) among mono-infected individuals by an estimated 0·81 (95% CI 0·14–1·49 higher) after adjusting for sex, which suggests that hookworm-infected individuals have significantly higher percentages of activated B cells compared with egg-negative individuals.

### Proliferative responses

**Figure 2:**
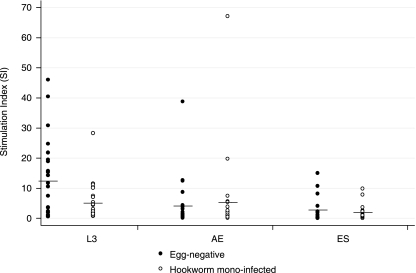
Proliferation of PBMCs, expressed as stimulation indices (SI), among egg-negative and hookworm mono-infected individuals after stimulation with different stage-specific hookworm antigens (L3, AE and ES); range and mean SI.

PBMCs from mono-infected individuals had significantly lower proliferative responses (*P* < 0·01) after *in vitro* stimulation with L3 antigen than egg-negative individuals (Figure 2), although this difference in proliferative response differed significantly between males and females (*P* = 0·04). Among females, SI was on average 11·06 lower (95% CI 5·60–18·41) among individuals with hookworm infection (*P* < 0·001), whereas among males, it was on average 2·56 lower (95% CI 3·34–8·45), however, this was not statistically significant (*P* = 0·40). The reduced proliferative responses to ES products and PHA observed among mono-infected individuals were not significantly different (*P* = 0·41 and *P* = 0·15, respectively).

### Cytokine and chemokine responses

Table 3 details mean cytokine responses by infection status. Overall, mono-infected individuals secreted lower levels of cytokines/chemokines in response to hookworm antigen stimulation than egg-negative individuals, with the exception of TNF-α after L3 and AE stimulation. This pattern was also seen among unstimulated control cultures (except IFN-γ) and after stimulation with PHA (except IL-13). Table 3 also shows that among mono-infected individuals, mean cytokine levels were lowest after stimulation with ES products, whereas after L3 stimulation a considerable secretion of cytokines was induced. Among egg-negative individuals there was a less distinguishable pattern of cytokine/chemokine responses. Values for IL-2 and IL-4 secretions were low for both infected and noninfected patients and without significant differences.

**Table 3:** Mean (standard deviation) cytokine and chemokine levels (pg/mL) in hookworm mono-infected and in egg-negative individuals

		Mean (SD) pg/mL			
		
Cytokine/chemokine	Antigen^a^	*n*	Egg-negative	*n*	Hookworm mono-infected
IFN-γ	Control	20	615·9 (1127·80)	23	700·8 (2614·48)
	PHA	20	11 249·9 (13 924·30)	26	5730·0 (13 072·02)
	L3	20	1610·6 (2242·96)	23	752·9 (1906·81)
	AE	20	1745·8 (2503·17)	23	586·6 (1405·16)
	ES	15	198·7 (276·95)	21	98·9 (137·00)
CXCL10	Control	25	90·5 (93·43)	26	78·3 (105·95)
	PHA	20	3038·3 (4402·01)	26	1424·6 (2030·63)
	L3	25	232·0 (502·54)	26	135·7 (288·87)
	AE	24	106·1 (172·84)	26	103·1 (210·34)
	ES	17	39·9 (77·75)	23	14·3 (29·21)
TNF-α	Control	25	292·4 (588·86)	26	456·4 (1864·94)
	PHA	19	2493·9 (2471·38)	26	2599·4 (4282·05)
	L3	25	185·4 (271·66)	26	322·8 (1309·80)
	AE	24	144·5 (201·59)	26	175·8 (665·90)
	ES	17	169·4 (185·14)	23	48·8 (69·15)
IL-13	Control	25	166·6 (90·98)	26	106·1 (92·09)
	PHA	20	530·4 (947·56)	26	824·5 (1188·55)
	L3	25	234·7 (148·11)	26	169·1 (138·00)
	AE	24	188·0 (114·29)	26	154·3 (116·90)
	ES	17	121·1 (76·76)	23	82·7 (94·18)
IL-5	Control	20	0·9 (3·80)	23	0·7 (2·45)
	PHA	20	368·3 (772·86)	26	287·5 (353·65)
	L3	20	170·5 (345·52)	23	136·9 (300·24)
	AE	20	222·2 (610·50)	23	86·3 (152·56)
	ES	15	77·7 (291·97)	21	44·0 (91·17)
IL-10	Control	20	458·5 (622·42)	23	237·0 (549·19)
	PHA	20	803·8 (1261·34)	26	283·7 (681·75)
	L3	20	579·0 (422·05)	23	429·7 (473·97)
	AE	20	247·7 (214·47)	23	172·6 (284·07)
	ES	15	614·7 (564·77)	21	170·5 (260·30)

Notes:

a PHA stimulated cytokine/chemokine responses after day 2 of stimulation; CXCL10, IL-13 and TNF-α after day 4 of stimulation; IFN-γ, IL-10 and IL-5 after day 6 of stimulation.

### Immune response to third-stage larvae

Using PCA, cytokine responses to L3 stimulation were reduced into three PC which explained 73% of the total variation in the data (Table 4). PC1 contrasts IL-5 and IL-13 levels (Th2 response) with TNF-α, IFN-γ and IL-10 (Th1/T regulatory response). However, no significant difference resulted in the mean component score between the two groups after adjusting for sex (*P* = 0·93); that is, there was no evidence that mono-infected individuals had a more skewed Th1/Treg or Th2 immune response than egg-negative individuals (Table 5). PC2 reflects a contrast between CXCL10 levels and the other cytokine responses, while PC3 reflects only CXCL10 levels. After adjusting for age and sex, the mean PC2 score was significantly lower among mono-infected individuals; that is, mono-infected individuals had a lower mixed immune response to L3 antigen than egg-negative individuals. There was no evidence that CXCL10 levels (mean PC3 score) differed significantly between egg-negative and mono-infected individuals (*P* = 0·09, see Table 5).

**Table 4:** Principal components analysis applied separately to cytokine responses after each antigen stimulation from different developmental stages of hookworm and among unstimulated control cultures

		Principal component^a^^b^		
		
Antigen stimulation	Cytokine/chemokine	1	2	3
Unstimulated controls	CXCL10	0·257	**0·491**	**–0·540**
	TNF-α	**0·560**	–0·183	0·063
	IFN-γ	**0·498**	0·105	0·074
	IL-10	**0·607**	–0·047	0·112
	IL-5	–0·060	**0·727**	–0·109
	IL-13	–0·010	**0·430**	**0·821**
L3	CXCL10	–0·027	–0·152	**0·983**
	TNF-α	**0·544**	0·289	–0·010
	IFN-γ	0·229	**0·490**	0·092
	IL-10	**0·569**	0·231	0·120
	IL-5	**–0·471**	**0·407**	0·068
	IL-13	–0·323	**0·659**	0·078
AE	CXCL10	0·004	**0·478**	**–0·569**
	TNF-α	**0·623**	–0·194	0·140
	IFN-γ	0·398	**0·411**	–0·378
	IL-10	**0·643**	–0·127	0·141
	IL-5	–0·128	**0·432**	**0·612**
	IL-13	0·153	**0·602**	0·346
ES	CXCL10	**0·512**	–0·149	NA
	TNF-α	0·033	**0·674**	NA
	IFN-γ	**0·544**	0·056	NA
	IL-10	–0·050	**0·677**	NA
	IL-5	**0·506**	–0·086	NA
	IL-13	**0·427**	0·234	NA

Notes:

a The figures represent the relative loading that each response contributes to each component. Strong loadings are indicated in bold

b Principal components 1 to 3 explained 73% of the total variation in the cytokine responses after L3 stimulation and in unstimulated controls, and 77% of the total variation after stimulation with adult worm antigen (AE); NA, not applicable. Only two components were needed to explain 73% of the total variation in cytokine responses after ES stimulation. The third principal component contributed less than the average of all cytokine responses (eigenvalue < 1).

**Table 5:** Estimated effect of infection status on mean score of cytokine responses after stimulation with antigens from different developmental stages of hookworm obtained by principal components; mean difference (95% CI) in scores by infection status adjusted for age and sex

	Component 1		Component 2		Component 3	
			
Antigen adjusted for	Mean difference^a^ (95% CI)^b^	*P*-value^b^	Mean difference (95% CI)	*P*-value	Mean difference (95% CI)	*P*-value
Unstimulated controls^c^						
Crude	0·18 (–1·74, 1·06)	0·84	0·38 (–0·99, 1·04)	0·54	0·41 (0·05, 1·03)	0·09
Sex	0·35 (–0·48, 1·27)	0·57	–0·52 (–2·01, –0·64)	0·14	0·34 (–0·20, 1·01)	0·28
L3						
Crude	–0·08 (–0·64, 0·54)	0·80	0·22 (–1·01, 0·78)	0·57	0·40 (–0·02, 1·15)	0·15
Sex	–0·04 (–0·61, 0·58)	0·90	0·77 (0·28, 1·45)	< 0·01	0·57 (0·15, 1·37)	0·05
Sex + Age	–0·03 (–0·66, 0·59)	0·93	0·74 (0·23, 1·31)	< 0·01	0·56 (0·12, 1·35)	0·09
AEc						
Crude	0·41 (–0·06, 1·11)	0·17	0·66 (0·22, 1·27)	< 0·01	0·21 (–0·19, 0·95)	0·45
Sex	0·56 (–0·002, 1·12)	0·05	0·88 (0·23, 1·40)	< 0·005	0·19 (–0·43, 0·91)	0·60
ES						
Crude	0·84 (0·17, 2·52)	0·09	1·15 (0·21, 1·99)	0·02	NA	NA
Sex	1·04 (0·12, 1·88)	0·02	1·38 (0·68, 2·29)	< 0·005	NA	NA
Sex + Age	0·91 (0·63, 1·66)	0·04	1·38 (0·64, 2·24)	< 0·005	NA	NA

Notes:

a Mean difference = mean principal component score for uninfected individuals – mean principle component score for those with a hookworm mono-infection

b 95% CI (confidence interval) and significance levels calculated by bootstrapping, using accelerated bias correction (10 000 replicates)

c There was no evidence of a relationship between age and mean principal component score among unstimulated controls or after AE stimulation and hence analysis was not adjusted for age; NA, not applicable. Only two principal components were needed to explain 73% of the total variation in the cytokine responses to ES stimulation.

### Immune response to adult worm somatic antigen

Cytokine responses after stimulation with AE antigen were also reduced into three PC that accounted for 77% of the total variation in the data (Table 4). PC1 reflects primarily TNF-α, IFN-γ and IL-10 responses. After adjusting for sex, the mean PC1 score was significantly lower among hookworm-infected individuals (*P* = 0·05); thus, egg-positive patients had significantly lower levels of TNF-α, IFN-γ and IL-10 when compared with egg-negative individuals. PC2 reflects a contrast between TNF-α and IL-10 production with CXCL10, IL-5, IL-13 and IFN-γ responses. There was also a significant difference in mean PC2 score between the groups after adjusting for sex (*P* < 0·005) such that mono-infected patients had significantly lower levels of CXCL10, IL-5, IL-13 and IFN-γ than TNF-α and IL-10 compared with egg-negative individuals (see Table 5). There was no significant difference in mean PC3 score between mono-infected and egg-negative individuals.

### Immune response to adult worm excretory/secretory products

Only two PCs were required to explain 73% of the total variation in cytokine responses after stimulation with ES products. PC1 reflects a mixed Th1/Th2 response (CXCL10, IFN-γ, IL-5 and IL-13), while PC2 reflects a contrast between CXCL10 on the one hand and TNF-α, IL-10 and IL-13 on the other. There was evidence that mean PC1 and PC2 scores were both significantly lower among mono-infected individuals (*P* = 0·04; *P* < 0·005, respectively). Therefore, mono-infected individuals show a lower mixed Th1/Th2 response and lower levels of TNF-α, IL-10 and IL-13 in response to ES antigen than egg-negative individuals.

## DISCUSSION

Here we show that the type and magnitude of the cellular and cytokine responses in *N. americanus* infected individuals differs for antigens from larval and adult stages of infection, as well as for somatic or secreted types of crude antigen extracts. While other studies have concluded that hookworm infection induces either a ‘down-regulated’ or a ‘highly activated immune system’, we demonstrate that both types of immune response occur during hookworm infection. Several features of our study permitted a robust investigation of these issues. First, we focused on individuals mono-infected with *N. americanus*to reduce potential confounding from other helminth co-infections. We compare them to a group of individuals who are egg-negative but resident in an area of high hookworm transmission and have therefore probably been exposed. We also sought to gain insight into the infection process by using crude antigen extracts from different developmental stages of the parasite within the host (i.e. larval and adult), as well as somatic and ES extracts from adults. Finally, we performed multivariate analyses using PCA to reduce the immunological variables to two or three different linear combinations of all the variables, known as principle components, accounting for most of the variability in the data. PCA is a widely used data-reduction technique (26), which is appropriate for highly skewed immune response data (25) and has been used extensively for the analysis of the immune response to soil-transmitted helminth infections (see for example (27,28)). After running PCA, each component may be given a biological interpretation according to the pattern of scores obtained, thus allowing significant trends to be determined without multiple testing.

T-cell responses play a major role in the expulsion of different gastrointestinal helminths in laboratory animals (29–31) and humans (27,28). Our data show that mono-infected individuals had lower CD3^+^ and CD4^+^ T-cell, and CD19^+^ B-cell counts, with only minor differences in CD8^+^ T-cell counts. These findings correspond with those from a hookworm-infected population in Nigeria (13). Another study however, showed decreased levels of CD4^+^ T-cells but increased number of CD8^+^ T cells among hookworm-infected individuals (14). This difference may be attributable to the fact that these individuals presented with multiple helminth and/or protozoan infections.

Although in the current study, hookworm-infected individuals had reduced T- and B-cell populations compared to egg-negative individuals, PCA provided evidence that activation of CD19^+^ B-cells, and early and late activation markers on CD3^+^ and CD4^+^ T-cells as well as the expression of HLA-DR on CD8^+^ T-cells were significantly higher among infected individuals. The increased activation of B-cells in infected individuals correlates with higher levels of IgG1, IgG4 and IgE against the three crude antigen extracts (data not shown). The higher levels of early (CD69) as well as late (HLA-DR) T-cell activation markers in mono-infected individuals are most likely induced by continuous antigenic challenge and ongoing infection from residence in an area of high hookworm transmission. The predominance of this cell phenotype can be gradually reversed by chemotherapy and elimination of existing helminths (14). Furthermore, the down-regulation of CD28 co-stimulatory molecules on CD8^+^ T-cells from helminth-infected individuals was thought to be an important factor contributing to the observed reduction in cellular reactivity (14).

Even though the graphical illustration of percentages of CD8^+^/CD28^+^ cells showed no clear differences between the patient groups, we did observe lower mean percentages of CD8^+^/CD28^−^ cells in mono-infected patients, and PCA analysis revealed a significantly lower score, for the PC largely weighted by CD8^+/^CD28^+^ and CD8^+^/CD28^−^ cells, in hookworm-infected individuals. Recently, it was shown that this cell population showed suppressive activity in an antigen-specific and a nonantigen-specific manner and therefore was denominated as CD8^+^ T suppressor lymphocytes (Ts) (32,33). CD8^+^/CD28^−^ cells derive from prolonged stimulation of CD8^+^/CD28^+^ cells and may be considered as T lymphocytes that have already undergone repeated activation, likely due to antigen stimulation. There are supposed to be three subsets of CD8^+^ Ts cells. A first type acts in an antigen-dependent manner via the transfer of inhibitory signals to antigen-presenting cells by direct cell–cell contact. A second subset of cells does not require antigen recognition and works via cytokine secretion, while a third type of cells are antigen-specific and act through IL-10 secretion (for review see (33)). The observed differences in percentages of CD8^+^/CD28^−^ cells between the patient groups and the results from PCA indicate that this cell population might play an important role in the regulation of the immune response during hookworm infection. Further experiments on the phenotype of CD8^+^/CD28^−^ cells in hookworm patients, their cytokine secretion pattern, and their *in vitro* activity can help to clarify their possible contribution in down-modulating the host immune response.

Other mechanisms of immune modulation during helminth infections can include regulation by co-stimulatory molecules on lymphocytes such as CTLA-4 (15), the expansion of regulatory T-cells (T_reg_), or an overall change in cytokine pattern. In experimental trematode and nematode infections, T_reg_ cells have been shown to play a pivotal role in the down-modulation of cellular reactivity and the inhibition of potentially protective immune responses (34,35). In our study, apart from higher levels of activation markers, mono-infected individuals had a significantly higher mean score of PC 2, which included CD4^+^/CD25^+^ T-cells (i.e. T_reg_-cells (36)). This shows the possible importance of this cell population in redirecting the immune response during hookworm infection. Though T_reg_ cells contribute to a significant degree in CD4^+^/CD25^+^ cells, intracellular staining of the transcription factor Forkhead box P3 (Foxp3) would help to better characterize this population of regulatory T cells (37). Also, expression of CTLA-4 on these CD4^+^/CD25^+^ T-cells was not analysed and further experiments are needed to elucidate the role of this cell population during hookworm infection.

Studies have shown diminished antigen-specific T-cell responses in PBMCs from helminth-infected individuals, who lack *in vitro* proliferation and have an altered cytokine pattern (7). Indeed, we have previously shown an altered immune response against parasite-specific and unrelated antigens in hookworm infected individuals (9). We add to this line of evidence the idea that levels of *in vitro* proliferation may differ according to the stage and type of crude antigen extract. Hookworm-infected individuals showed significantly lower proliferative responses to L3 antigen, as well as lower responses to ES or to PHA. Hamsters experimentally infected with hookworm have shown a similar down-modulation of proliferative responses after stimulation with hookworm antigens or mitogen; however, this down-regulation becomes evident only after the onset of patency and not during the initial phase of larval migration (38), which implies that the mechanisms leading to the reduced cellular reactivity come with established, adult infection and are most probably induced by their ES products. We believe that this reduced cellular reactivity is a mechanism of inhibiting larval attrition; that is, incoming larvae are not killed in the skin or the lung, but survive to the gut as shown by others (39). Further proof is that this state of reduced cellular responsiveness can be reversed in part by anthelminthic chemotherapy (9,10).

With the exception of TNF-α, cytokine and CXCL10 concentrations were lower in mono-infected individuals, a pattern also observed in unstimulated cultures and after stimulation with mitogen. However, the total variation in cytokine responses differed depending on the hookworm antigen used for stimulation, suggesting that each developmental stage of hookworm induces a different immune response, with no distinct split into a Th1 or Th2 profile. Such stage-specificity in the immune response has been described for other human helminth infections (40–42). After L3 stimulation, mono-infected individuals had lower levels of IFN- γ, IL-5 and IL-13. This mixed Th1/Th2 profile reflects the positive association between IFN-γ and IL-5 responses after L3 antigen stimulation among hookworm-infected individuals and contradicts the widespread opinion that IFN-γ has an antagonistic effect on Th2 responses. However, this mixed profile was not seen in egg-negative individuals, where a negative association between IFN-γ and IL-5 secretion was observed. After stimulation with AE and ES antigens, IFN-γ, IL-5 and IL-13 responses, in addition to CXCL10, were significantly lower in mono-infected individuals. Such a mixed immune response in hookworm-infected individuals has already been described, albeit at diminished cytokine concentrations (8–10). CXCL10 is supposed to play an important role during inflammatory processes (Th1). On the other hand, CXCL10 secretion is blocked by IL-4 or IL-10. Most recently, it was shown that CXCL10 is modulated during experimental *T. muris* infection. In susceptible animals, CXCL10 is induced locally and slows the ‘epithelial escalator’, resulting in crypt-cell hyperplasia, an increase in the epithelial niche, and survival of parasites in the intestinal tract (43). However, in chronically infected hookworm patients, systemic Th1 immune responses have already been down-modulated. There was no evidence that elevated CXCL10 production contributed to the survival of adult and attached worms in the intestinal tract. A recent experimental human infection resulted in elevated systemic secretion of IFN-γ, IL-5 and IL-13 during the initial phase of infection until early patency, after which these cytokines were down-modulated, coincident with the establishment of the adult worms and initiation of egg deposition in the intestinal tract (44).

After stimulation with ES products, cytokine secretion – with the exception of IL-5 – remained lower in both groups than in unstimulated PBMC cultures, demonstrating the down-modulating potential of secretions from adult worms. In addition, production of TNF-α and IL-10 after stimulation with ES products differed by infection status, with hookworm-infected individuals producing significantly less, which might serve as a mechanism to minimize intestinal inflammation at the site of worm attachment in infected patients. In contrast, after stimulation with L3 or AE antigen, TNF-α production did not differ significantly by infection status. Until now, however, only one ES product has been studied in detail for its potential of immunomodulation and has been shown to bind selectively to human NK-cells and induce secretion of IFN-γ (45).

In summary, we have shown that adults infected with *N. americanus* have reduced but activated T-cell populations and show a differential pattern of proliferative responses to stage-specific antigens, with responses to L3 antigen being significantly lower than in egg-negative individuals. Furthermore, the secretion of cytokines and chemokines and the observed ‘mixed’ Th1/Th2 immune response to somatic worm antigens was significantly lower in hookworm-infected individuals. Finally, ES products from adult worms were shown to be a potent down-modulator of cytokine and chemokine responses, especially of inflammatory TNF-α secretion, which possibly serves as a mechanism of immune evasion that prolongs survival of the helminth in the human host. The described changes in the immune system during hookworm infection are most likely induced by a set of different stage-specific antigens, possibly secreted products, which keep the patient in a state of reduced cellular responsiveness, such that the parasite is able to survive in a ‘happy valley’ of ‘mixed’ Th1/Th2 immune responses with reduced intestinal inflammation. Repeated treatment with anthelmintics (10) or the future development and application of a hookworm vaccine (46) are possible tools that could put the human host in a more favourable position to counterattack these harmful parasites.
